# Hedonics Act in Unison with the Homeostatic System to Unconsciously Control Body Weight

**DOI:** 10.3389/fnut.2016.00006

**Published:** 2016-02-15

**Authors:** Heike Münzberg, Emily Qualls-Creekmore, Sangho Yu, Christopher D. Morrison, Hans-Rudolf Berthoud

**Affiliations:** ^1^Pennington Biomedical Research Center, Louisiana State University System, Baton Rouge, LA, USA

**Keywords:** obesity, body weight, eating behavior, brain, food reward, palatability, cognitive control, decision-making

## Introduction

With the global obesity crisis continuing to take its toll, the demand for solutions has increased. The discussion about nature vs. nurture and biology vs. psychology has culminated in declaring obesity as a disease by some medical organizations. Environmental factors and genetic predisposition, rather than personal responsibility are to blame, as for any other disease. This view implies that the biological processes regulating body weight are essentially operating at the unconscious realm. Although this has long been accepted for the so-called homeostatic regulation of energy balance, it is less clear for the hedonic controls. Here, we critically evaluate the important question how rodent models can help understand the contribution of hedonic neural processes to body weight regulation. When looking at the concepts of reward, reinforcement, motivation, pleasure addiction, and their neural mechanisms, in the context of eating and exercise, the new view emerges that homeostatic and hedonic controls are closely interrelated and often act in unison at the unconscious level to achieve biologically adaptive responses. Although the discussion of a body weight set point has been neglected in recent years, this topic becomes more pressing as an important aspect for effective treatment of obesity.

## Hedonic Mechanisms Overpower Homeostatic Regulation

When the body weight of animals and humans is disturbed by periods of either under- or overfeeding, it promptly returns to pre-perturbation levels through a process termed homeostatic regulation that involves the controls of both energy intake and energy expenditure (1, 2). The basic hypothalamic circuitry underlying this regulation has long been known (3) and was much refined, particularly over the last 20 years in the wake of the discovery of leptin. In brief, two distinct neural populations in the mediobasal hypothalamus act as primary energy sensors and engage a complex network of effector circuits controlling both energy-in and energy-out in a biologically adaptive fashion [for review, see Ref. (4–7)].

However, while most agree with such basic homeostatic regulation, there has been much ­discussion regarding the exact level of defended body weight and the mechanisms involved (8–13). Clearly, there is no fixed set point around which mammalian species regulate their body weight. Rather, it is flexible, depending on both internal and external conditions including genetic and epigenetic predisposition, food availability, food palatability, and other environmental factors (10). This is best illustrated by the seasonably variable and homeostatically defended body weight set point of hibernators (14).

**Figure 1:**
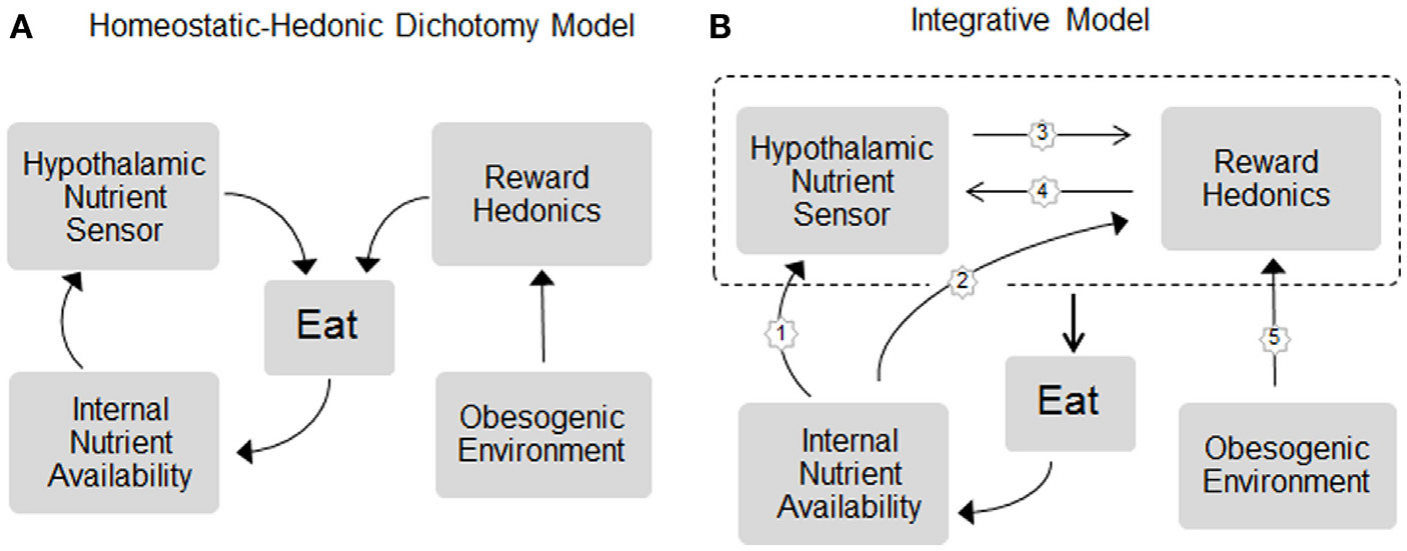
**Schematic representation of the (A) dichotomy and (B) integrative models of homeostatic and hedonic control of food intake and regulation of body weight**. In the dichotomy model, homeostatic and hedonic mechanisms are largely independent. In the integrative model, the neural circuitries for internal nutrient sensing and hedonic processing act in concert to control eating and body weight and the hedonic system becomes part of the homeostatic system. Signals of both short- and long-term internal nutrient availability, such as leptin, gut hormones, and metabolites, are sensed by both, the hypothalamic nutrient sensor (pathway 1) and hedonic/reward processing centers (2), and these circuits communicate bidirectionally with each other (3 and 4) to unconsciously affect eating. For simplicity, energy expenditure as effector mechanism in the regulation of body weight is not included. For details, see text.

One factor that is widely believed to be very important for influencing the individual body weight set point is food hedonics, particularly the shift toward higher body weight by highly palatable, calorie-dense foods (Figure 1A). The clearest example of this shift in defended body weight is the cafeteria diet-induced obese rat and mouse (15). Although it is suspected that the increased availability of highly palatable, energy-dense foods is also mostly responsible for the current obesity epidemic, it is much harder to prove, because of difficulties to strictly control energy balance and environmental conditions in humans over extended periods of time as it is possible in animal models. A widely accepted view is that in genetically and/or epigenetically susceptible individuals, the obesogenic food environment is able to establish a new, higher body weight set point that is similarly defended against forced fasting and overfeeding as in normal weight individuals (11). Therefore, one of the key issues in understanding body weight regulation is the neurological explanation for this shift in defended body weight. What are the neural mechanisms that allow availability and palatability of energy-dense foods to overpower the basic homeostatic defense system? Understanding these mechanisms could lead to the development of more specific drugs or behavioral interventions in the fight against obesity.

## Hedonic Processing is an Integral Part of the Homeostatic Regulatory System

The view that the hedonic and homeostatic neural circuitries are not separate entities but are part of the same regulatory system is rapidly gaining traction. This is based on evidence for bidirectional modulation of corticolimbic brain areas by interoceptive signals, and of the hypothalamus by exteroceptive signals and their cognitive and emotional correlates (Figure 1B).

### Bottom-up Modulation of Corticolimbic Circuits of Cognition and Motivation by Interoceptive Signals of Nutrient Availability

The bottom-up control of hedonic and cognitive processes by internal signals is not a new insight. Given the crucial importance of nutrients for survival, it is a fundamental attribute of the expression of hunger and goes back to the beginning of evolution of the nervous system. Specifically, the hungry state is characterized by increased incentive salience attribution (the mechanism by which a goal object such as food is becoming highly desired and wanted – a behavioral magnet), which is neurologically manifested by heightened activity of the mesolimbic dopamine system (16–18). What is new, are some of the messengers and neural mechanisms shown to be involved. For example, it is now clear that one of the most eminent homeostatic regulators of body weight – leptin – modulates appetite by acting not only on the hypothalamus but also on components of the mesolimbic dopamine system (19–22) and on olfactory and taste sensory processing (23–25). Similarly, many other internal signals of nutrient availability, such as ghrelin, intestinal GLP-1 and PYY, and insulin, as well as glucose and fat, also partly act on corticolimbic structures involved in the cognitive and rewarding aspects of food intake control (26–36). Effects on cognitive functions by these hormones are interesting in the context of human studies showing impairments of both cognitive and metabolic functions in obese patients (37–39). Although the common link is not yet known, a leading hypothesis suggest that intestinal dysbiosis resulting from an interaction between sub-optimal nutrition, gut microbiota, and the innate immune system with subsequent changes in gut-to-brain signaling and blood–brain barrier integrity are important (40–43).

### Top-down Modulation of the Classical Hypothalamic Regulator by Sensory, Cognitive, and Motivational Signals

The other driver of this integrated view is new insight into the top-down modulation of classical homeostatic circuitries by cognitive and emotional processing in corticolimbic systems (44). Cue-induced, conditioned food intake is thought to be an important mechanism in overeating by humans in an obesogenic environment (45, 46) and has been studied in rodents for quite some time (47). Some of the relevant pathways involved in this cognition-dependent food intake have been identified in the rat by demonstrating dependence on amygdala and prefrontal cortex-to-lateral hypothalamus projections (48, 49). Most recently, evidence for top-down modulation of AGRP neurons in the mediobasal hypothalamus, the epicenter of classical homeostatic regulation, was presented. These powerful neurons have been thought to be mainly controlled by circulating hormones and metabolites in a relatively slow waxing and waning fashion commensurate with the fasted and fed states. Using modern, genetically based neuron-specific technology, it was demonstrated that activity of AGRP neurons is also controlled on a second-by-second basis by the conditioned expectation of imminent food ingestion (50, 51). This acute external sensory and cognitive control over AGRP neuron firing rate is likely accomplished by direct or indirect inputs from a number of cortical and subcortical areas as demonstrated by neuron-specific retrograde viral tracing (52).

## Control of Food Intake and Regulation of Energy Balance is Predominantly Subconscious

It is clear that the classical hypothalamic neural circuitry responsible for the homeostatic regulation of energy balance and body weight, similar to homeostatic regulation of other bodily functions, such as blood glucose or blood pressure, is operating largely beyond awareness, at the unconscious level. In addition and as discussed above, the incentive sensitization mechanism by which interoceptive signals of energy depletion such as low leptin drive “wanting” through the mesolimbic dopamine system (16, 18, 53) is also largely operating outside awareness as demonstrated in human neuroimaging studies (54–56). Even in the absence of metabolic hunger and associated interoceptive sensitization signals, conscious awareness of the cue does not seem necessary. This has been shown in rats with cue-induced conditioned food intake (47, 48). Furthermore, the human brain can learn the value of monetary rewards and use it for decision-making without conscious processing of contextual cues (57). Although optimal decision-making requires self-control, represented in the dorsolateral prefrontal cortex (58, 59), the transformation of reward-driven behavioral action is not under obligatory control of this brain area and often constrains the free will to act (60). Finally, neural activity in certain brain areas can be going on for quite some times before humans become aware of their own decision (61, 62), suggesting that much of the processes leading to a decision are taking place at the unconscious level.

Ingestive behavior in both humans and rodents appears to become particularly resistant to cognitive controls when it is highly habitual (63, 64). Under normal conditions, information about possible outcomes is important for cue-induced goal-directed actions making such actions sensitive to devaluation. However, habitual behavior no longer depends on learned reward expectations and is thus largely insensitive to mechanisms of reward devaluation (64, 65). The neural circuits governing non-habitual behaviors are differently organized than those for habitual or automatic behaviors. Non-habitual behaviors heavily depend on the ventral striatum (nucleus accumbens) and the ventromedial prefrontal cortex, whereas habitual behaviors depend more on the dorsolateral striatum (65, 66). The memory storage and recall mechanisms are also different for habitual vs. non-habitual actions and behaviors. In distinction to declarative memories which require a conscious mind, procedural memories operate largely below the level of conscious awareness and storage is more distributed (67–69). As a consequence, procedural memories and the habitual ingestive behaviors they guide are relatively resistant to inhibitory cognitive control and executive functions.

## Conclusion

Animal models have been crucial for dissecting the complex mechanisms underlying predisposition to obesity. Given that the overwhelming majority of genetic loci linked to human obesity are associated with neural functions (70), it is not surprising that the neural controls of food intake and regulation of energy balance are a main component of these mechanisms. Although functional neuroimaging in humans is also starting to make important contributions, only the more invasive approaches in rodents have been able to provide mechanistic explanations. As a result, the traditional dichotomy between homeostatic and non-homeostatic/hedonic systems responsible for the control of appetite and regulation of body weight, although heuristically still useful, no longer adequately describes the extensive anatomical and functional interactions between the two systems. In addition, much of the output of this larger interactive system is bypassing awareness. The implications of these new insights are far reaching as they will guide not only future research but also the design of pharmacological and behavioral therapies for obesity and eating disorders.

## Author Contributions

HM and CM helped conceive the opinion, reviewed the literature, wrote parts of the manuscript, and edited the pre-final version of the manuscript. EQ-C and SY were involved in discussions of the original idea, reviewed parts of the literature, wrote parts of the manuscript, and edited the pre-final manuscript. H-RB conceived the original idea for the opinion, discussed several draft versions of the manuscript with all coauthors, researched the literature, and wrote the final manuscript.

## Conflict of Interest Statement

The authors declare that the research was conducted in the absence of any commercial or financial relationships that could be construed as a potential conflict of interest.
